# Peer Review of “Investigating the Variable Component of the Systematic Error, a Neglected Error Parameter: Theoretical Reevaluation Study”

**DOI:** 10.2196/88830

**Published:** 2026-02-27

**Authors:** Elvar Theodorsson

**Affiliations:** 1 Linköping University, Linköping, Sweden

**Keywords:** repeatability condition, reproducibility within laboratory condition, measurement, systematic error, clinical laboratory, quality control, bias, QC, statistical, statistics, mathematics, computer simulation, standard deviation

*This is the peer-review report for “Investigating the Variable Component of the Systematic Error, a Neglected Error Parameter: Theoretical Reevaluation Study.”*

## Round 1 Review

The phenomenon the author of this paper [1] calls “the variable component of the systematic error” (VCSE) is highly important and relevant, and the approach proposed by the author is worthy of serious scientific dialog. However, a prerequisite for serious dialog is (1) a well-structured manuscript based on (2) extensive knowledge of the state of the art in calculating measurement uncertainty and (3) well-written English text.

Unfortunately, this manuscript fails in all three aspects. As a reviewer, I urge the author to seek collaboration with a scientist even better versed in the actual field(s) than himself to create a more deserving manuscript.

1. The handling of constant or intermittent bias has been a challenge for more than 200 years, especially since Gauss and Laplace’s work in the early 19th century. The author refers to Eisenhart’s excellent 1963 paper, which is appropriate but not as the origin of VCSE. Shewhart’s 1923 and 1939 books [2,3] also address this matter.

2. Bias, including VCSE, is also a major point of contention in the International Bureau of Weights and Measures/International Organization for Standardization work on the Guide to the Expression of Uncertainty in Measurement and its revision. The complexity of the issues is illustrated, for example, in a book by Krystek [4]. The current manuscript illustrates the opinions of its author but fails to illustrate the background of the immense scientific literature and debates that have already dealt with the matter.

3. The author needs to clarify whether he adheres to the error or uncertainty paradigms in measurement uncertainty/error questions. The current manuscript represents a mixture of both.

4. The “thought-provoking and even shocking” fact that the Westgard rules and power calculations are based on repeatability uncertainty and not on reproducibility uncertainty is well-known by metrologists in clinical chemistry. Unfortunately, mentioning this fact commonly hurts the sentiments of a majority in our field, and many of us avoid harping on it. A prerequisite for appropriately using Westgard rules and variants is that changing goal mean values are used as calibrators, and reagent lots change over time.

5. The traceability hierarchies used when producing reference materials and calibrators are usually claimed to explain the variations experiences (eg, during lot-number changes). The author apparently does not accept this explananation of the main cause of lot-number shifts/bias, and he needs to explain why his mathematical/statistical theory should be accepted instead.

6. In a crucial part of his manuscript, the author claims that “While RE changes unpredictably from measurement to measurement, VCSE(t) [variable component of systematic error at the moment t] remains quasi-constant in a given day, influencing all measurement results obtained in that day systematically. But in long-term experiments, VCSE(t) becomes a cyclical time-variable function, which repeats the same values after unequal periods. (A period may last even one month).” The author presents Cobas 6000 analyzer data in support of his thesis. However, data from a variety of measuring systems, lot changes, and measurands are needed before this theory of a cyclical phenomenon is chosen instead of a theory of random components.

7. The author’s approach deserves to be published in a better-structured manuscript, written in far better English than the English language of the present manuscript.

## Round 2 Review

In the first round of reviews, I asked for “(1) a well-structured manuscript based on (2) extensive knowledge of the state of the art in calculating measurement uncertainty and (3) well-written English text.”

The revised version of the manuscript has improved the English text but needs to improve in the two other aspects.

I agree that the paper’s subject is essential. The author is well-versed in mathematical statistics and has practical experience in laboratory quality control. However, the manuscript lacks in:

1. Counting in metrological aspects
2. Using a conventional manuscript structure
3. Showing sufficient real laboratory results and the consequence of using the proposed paradigm on real laboratory results

## Abbreviations

VCSE: variable component of systematic error
VCSE(t): variable component of systematic error at the moment t
